# The Gestational Diabetes Management System (GooDMomS): development, feasibility and lessons learned from a patient-informed, web-based pregnancy and postpartum lifestyle intervention

**DOI:** 10.1186/s12884-016-1064-z

**Published:** 2016-09-21

**Authors:** Wanda K. Nicholson, A. Jenna Beckham, Karen Hatley, Molly Diamond, La-Shell Johnson, Sherri L. Green, Deborah Tate

**Affiliations:** 1 Department of Obstetrics and Gynecology, University of North Carolina School of Medicine, Chapel Hill, NC USA; 2 Partnerships for Women’s Endocrine and Reproductive Health (PoWER), University of North Carolina School of Medicine, Chapel Hill, NC USA; 3 Diabetes and Obesity Core, Center for Women’s Health Research, University of North Carolina School of Medicine, Chapel Hill, NC USA; 4 The Diabetes Center, University of North Carolina School of Medicine, Chapel Hill, USA; 5 Cecil G. Sheps Center for Health Services Research, University of North Carolina School of Medicine, Chapel Hill, NC USA; 6 Department of Nutrition, University of North Carolina School of Medicine, Chapel Hill, USA; 7 Department of Health Behavior, University of North Carolina Gillings Global School of Public Health, Chapel Hill, USA; 8 Program on Obesity Treatment, University of North Carolina Gillings Global School of Public Health, Chapel Hill, NC USA

**Keywords:** Gestational diabetes, Behavioral intervention, Web-based intervention, Pregnancy, Postpartum, uhealth, mhealth

## Abstract

**Background:**

Gestational diabetes mellitus (GDM) contributes to the epidemic of diabetes and obesity in mothers and their offspring. The primary objective of this pilot study was to: 1) refine the GDM Management System (GooDMomS), a web-based pregnancy and postpartum behavioral intervention and 2) assess the feasibility of the intervention.

**Methods:**

In phase 1, ten semi-structured interviews were conducted with women experiencing current or recent GDM mellitus GDM to garner pilot data on the web based intervention interface, content, and to solicit recommendations from women about refinements to enhance the GooDMomS intervention site. Interviews were audiotaped, transcribed and independently reviewed to identify major themes with Atlas.ti v7.0. In phase 2, a single-arm feasibility study was conducted and 23 participants were enrolled in the GooDMomS program. Participants received web lessons, self-tracking of weight and glucose, automated feedback and access to a message board for peer support. The primary outcome was feasibility, including recruitment and retention and acceptability. Secondary outcomes included the proportion of women whose gestational weight gain (GWG) was within the Institute of Medicine (IOM) guidelines and who were able to return to their pre-pregnancy weight after delivery.

**Results:**

Comments from semi-structured interviews focused on: 1) usability of the on-line self-monitoring diary and tracking system, 2) access to a safe, reliable social network for peer support and 3) ability of prenatal clinicians to access the on-line diary for clinical management. Overall, 21 (91 %) completed the pregnancy phase. 15/21 (71 %) of participants were within the Institute of Medicine (IOM) guidelines for GWG. Sixteen (70 %) completed the postpartum phase. 7/16 (43 %) and 9/16 (56 %) of participants returned to their pre-pregnancy weight at 6 and 30 weeks postpartum, respectively.

**Conclusions:**

This study documents the feasibility of the GooDMomS program. The results can have implications for web technology in perinatal care and inform the current care paradigm for women with GDM. Findings are supportive of further research with recruitment of a larger sample of participants and comparison of the outcomes with the intervention and standard care.

**Trial registration:**

The study was registered at ClinicalTrials.gov on May 15, 2012 under protocol no. NCT01600534.

## Background

Gestational diabetes mellitus (GDM) is part of an endocrine and reproductive cycle that contributes to the epidemic of diabetes and obesity across generations. In utero alterations to fetal metabolism due to prolonged glycemic exposure have life-long consequences for the newborn; namely a 8-fold risk of diabetes as well as chronic obesity and its downstream sequelae. Also, the expectant mother with GDM is at greater risk for long-term obesity, overt diabetes [1,2] and the metabolic syndrome [3].

It may be possible to achieve weight and glucose control in women with GDM and reduce the risk factors for development of overt diabetes with behavioral modifications, but there is limited data on the effects of a combined pregnancy and postpartum behavioral intervention in women with GDM. Evidence from earlier studies of prenatal nutrition counseling [4], or late prenatal [5] or postpartum [5,6] curricula to prevent postpartum weight retention suggest that women with GDM can benefit from short-term interventions. The Diabetes Prevention Program (DPP) [7] included women with histories of GDM [8], but they were remote from diagnosis at the time of enrollment.

Women with GDM represent a unique population to test innovative methods to deliver theory-based behavioral interventions. First, women are not always fully aware of the effects of elevated glucose levels and excessive weight gain during pregnancy on their long-term health or the health of their offspring. Interventions tailored to address these specific gaps in knowledge are crucial to helping women make the lifestyle changes necessary for glucose and weight control. Despite the increased risk of type 2 diabetes, women with GDM often have competing demands of childcare, work and family commitments that make it difficult to adopt and maintain healthy lifestyle behaviors after delivery. Our goal was to use a mixed-methods approach to develop and test the feasibility of a patient-centered, web and text-based behavioral intervention to help women with GDM achieve weight and glucose control during pregnancy and postpartum. Alternative methods for delivering interventions, such as web-based technologies that are easily accessible to women with GDM and provide flexible contact patterns, should be considered.

We proposed a novel intervention that begins in pregnancy at the time of GDM diagnosis and extends seamlessly into the postpartum period and has both pregnancy and postpartum-specific maternal and fetal outcomes. A previous study examined the feasibility of a web-based intervention on increasing women’s knowledge [9]. Our objective was to develop an intervention to change behavior and take advantage of an important “teachable moment” [10] and offers the possibility of affecting the life course of both the mother and her developing fetus. In phase 1, we developed and refined the GooDMomS intervention incorporating feedback received from semi-structured interviews and qualitative analysis. In phase 2, we conducted a pilot study of the web-based pregnancy and postpartum intervention. The primary outcome was to assess feasibility, including recruitment and retention. Secondary outcomes included the proportion of women with gestational weight gain within the Institute of Medicine (IOM) guidelines [11], weight retention at 6 weeks and 30 weeks postpartum. Other outcomes included weekly rate of gestational weight gain, change in HbA1c levels and insulin resistance, as measured by the Homeostatic Model Assessment [12]. Because the offspring of women with GDM have an increased risk of obesity and diabetes as young adults, we also examined the feasibility of collecting neonatal measures of infant body composition.

## Methods

### Phase 1: development and refinement of the GooDMomS intervention

#### Content

Our gal was to introduce a lifestyle program integrated seamlessly across two critical periods in the lifespan of both mother and child-the prenatal period with the diagnosis of GDM and metabolic alteration that occur and the early postpartum period where maternal behavioral modifications can impact the long-term health of the mother and her offspring. The intervention provides evidence-based recommendations for weight and glucose information control combined with current clinical practice guidelines. The University of North Carolina Institutional Review Board (IRB # 12–0782) approved the qualitative study in phase 1 and feasibility study in phase 2.

#### Web and text components

We emphasized two aspects of social cognitive theory (SCT) [13], self-efficacy and self-regulation, to guide intervention development. GooDMomS was developed to encourage self-management and the ability of women to modify their lifestyle behaviors through structured goal setting, automated feedback tailored to each individual’s progress in weight, control, dietary intake and physical activity, self-monitoring and social support. GooDMomS was designed to promote self-management and self-regulation in weight and glucose control, including i) pregnancy and postpartum web lessons, ii) a web-based self-monitoring diary for participants to record their daily weight, exercise and glucose levels during pregnancy and weight, calories, and exercise during the postpartum period, iii) weekly healthy recipes and tips about maintaining a healthy lifestyle, iv) an online message board for peer support and to pose questions or concerns for the study’s interventionist and v) weekly text messages.

### Qualitative assessment

#### Design, recruitment, setting

We conducted 10 semi-structured interviews in English using a purposeful sample of women with current or recent GDM between January and April, 2012. We continued with interviews until saturation was achieved [14]. Participants had an average age of 33 years (range, 23–44 years). The racial composition of participants was four African Americans, four Caucasians and two Hispanic whites. All were attending university clinics for prenatal and postpartum care. Four participants were Medicaid recipients; seven participants were multiparous.

#### Procedure and analysis to assess ease of use and content

Participants attended a 30-min semi-structured interview. Interviews were conducted while women explored a computerized diabetes behavioral intervention. After obtaining written informed consent, each participant was asked a series of eight open-ended questions in English by a trained interviewer regarding the ease of use of the web components and suggestions were sought for recommended refinements. Question were posed to offer patient generated feedback regarding the most useful components and to suggest additions as they actively browsed the website and toggled back and forth to each of the components: 1) How easy was it to follow the layout of the web-based program, including font size, formatting of web lessons and clarity of the images, 2) Do the web lessons provide useful information, 3) Are there topics that we should add to the content of the web lessons, 4) How usable is the self-tracking page, 5) Is the personal progress report page useful?, 6) What are your thoughts about an online message board for communicating with other mothers with GDM, 7) What is the most useful section of the web-based program and 8) What is the least useful section of the program? The interviewer used reflective probes to encourage respondents to clarify and expand on their verbal comments. All interviews were audiotaped and reviewed independently by two reviewers (AJB, SLG) who coded each statement to identify salient themes. Additional codes for new and emerging concepts were based on a grounded theory approach [14] and organized into broad themes using Atlas.ti v6.2 (Berlin, Germany). A third investigator reconciled discrepancies. We continued to sample, conduct interviews, and analyze data until redundancy of information was obtained, at which time recruitment for interviews stopped.

#### Themes and representative quotes

Participant responses were categorized into 4 broad domains: 1) perceived ease of use of web components, 2) assessment of usefulness of web-based components, 3) attitudes about online peer support and 4) preferred components for a GDM clinical management program. Participants expressed interest in each of the website components.

##### Perceived ease of use

Most participants (*n* = 8) thought the website was user-friendly and easy to access.

“I thought it was concise. I mean, on the Web, me personally, you don’t want to sit there reading pages and pages, so I think it was organized well. [It] had the bullet points broken out so you can get the gist of it pretty quickly.”

##### Usefulness of web-based components

Participants verbalized that the link to healthy recipes and the “tracking your progress” page to record daily weights, exercise and blood glucose levels were the most useful website component.

“I will probably be using that every day,…*every day*, because it’s, I guess it’s quicker for me to jump on the computer and put it in than it is to write it in the book, because I’ll forget it.”

##### Attitudes about online peer support

Women reported little to no experience with online discussion groups, but expressed a willingness to use a message board to communicate with other women with GDM

“Using this program would probably…would be the first for me because I don’t do the message boards and things of that nature, but I’m willing to give it a try, just, you know, because somebody may know something more than I do, and it never hurts to ask.”

##### Preferred components of a web-based intervention

While each of the participants was pleased with the current features of the web program, they desired guidance for entrée selections at major restaurants so that they could maintain a healthy diet, while eating out.

“Like, maybe some examples from a typical restaurant, things that might be good on a menu to order at a restaurant…… because, I don’t know, I didn’t feel like cooking when I was pregnant

Also, participants suggested modifying the site to allow their prenatal providers to have direct access to the site to view their glucose and weight information.

It would be great if the doctors could actually have access to it or, you know, whoever [is] checking it for you.”

#### Feedback and refinement of intervention website

Comments from the semi-structured interviews were presented to the research team and used to refine the web-based intervention. The final version of the website used in the feasibility study in Phase 2 (www.goodmoms.org) was comprised of multiple features grounded in social cognitive theory [15]. We integrated additional cooking tips and recipes into the website and expanded the self-tracking page to show graphic representation of fasting glucose levels and weekly weights. To promote the use of the message board, the importance of social support was integrated into the pregnancy and postpartum web lessons. The web-based intervention included a pregnancy site for the prenatal period and a postpartum site for after delivery. Access to the website was limited to active participants who were assigned a login/password combination and interventionists.

Further refinements included the development of four animated videos to 1) provide a better understanding of the effects of GDM on fetal growth, 2) demonstrate physical activities that could be safely performed in pregnancy and 3) emphasize strategies to promote healthy lifestyle behaviors, including exercise and breastfeeding after delivery and 4) promote compliance with postpartum glucose testing. Animation was used to explain biological pathways that contribute to the development of GDM and affect fetal growth. Each video had a structured script and was narrated by a professional narrator.

### Phase 2: procedures for feasibility study

#### Study setting and design

We used a single arm, pretest-posttest study design in women diagnosed with GDM attending two university-based prenatal clinics and one community-based practice. One of the university-based clinics was located within the hospital and one was located within the surrounding community. The community office practice was located approximately 10 miles from the university. Each of the recruitment sites screens for GDM between 24 and 28 weeks of pregnancy using the two-step screening strategy recommended by American College of Obstetricians and Gynecologists which includes a 50 g, 1-h glucose challenge test, followed by a 100 g, 3-h oral glucose tolerance test in those with a 1-h value ≥ 140 mg/dl. An appointment with a nutritionist and diabetes educator are scheduled within one week of their diagnosis. Visits with their obstetricians usually occur at 2-week intervals. Glycemic targets are fasting levels < 95 mg/dl and 1-h levels < 140 mg/dl. If more than 40 % of the glycemic targets are not met in a given week, follow-up visits with the nutritionist and educator are arranged and women are placed on an insulin or oral agent.

Potential participants were referred for eligibility screening by the diabetes prenatal nurse at each of the recruitment sites. Per clinical protocol, the diabetes nurse contacts each patient by phone to inform them of their positive test results. Women who were diagnosed with GDM were provided with general information about the study during their calls with the diabetes nurses. If the patient expressed an interest in the study, her contact information was sent to the study recruiter who screened the participants for eligibility over the phone. If the participant was deemed eligible, the recruiter arranged to meet the potential participant at her next prenatal or nutrition/diabetes education visit to obtain informed consent, complete study enrollment and collect baseline data. We report this non-controlled intervention study using guidelines from Transparent Reporting of Evaluations with Non-Randomized Designs [16].

#### Study population

Women ages 21 years and older with newly diagnosed GDM were recruited between July, 2012 and April 2013. Additional inclusion criteria included women who were English speaking and had computer and internet access for personal use at least 8 h per week. Medical exclusion criteria included a history of chronic hypertension or pre-existing diabetes, recent epilepsy, cardiac event or stroke in last 6 months, and special nutritional needs (e.g. malignancy or other chronic disease).

### Intervention

The GooDMomS intervention was comprised of two phases: prenatal and postpartum. The intervention incorporated three domains, including knowledge and perceptions of risk for the mother and the fetus/infant, behavioral challenges and strategies, and social support and lifestyle modifications.

#### Pregnancy phase

The pregnancy phase of the intervention began within 2 weeks of the diagnosis of GDM and continued until 36 weeks of pregnancy. After enrollment and baseline data collection, participants were provided with a unique login and password to gain access to the website. Within 48 h of enrollment, participants received an introductory call from the team’s interventionist to address any technical problems, difficulties with login/password combinations and to respond to questions about the GooDMomS program. If a participant had not logged in within 1 week of enrollment, the interventionist called the participant to remind them to log into the program. During the first login of each program week, participants were posed a series of automated check-in questions that asked them to enter their weight and whether they had achieved their physical activity and dietary goals over the previous week. Current ACOG guidelines recommend at least 30 min or more of moderate exercise on most, if not all days of the week [17]. GooDMomS participants were encouraged to engage in 30 min of moderate exercise at least 5 times per week (150 min per week). If a participant did not achieve their goals, an additional question asked them to identify the barriers that may have contributed to not achieving the goal. The participant then received tailored readings to address the selected barriers.

In the pregnancy phase of the intervention, participants were given 6 web lessons (Appendix) presented over 6 weeks which were developed by the research team and informed by practice recommendations and clinical guidelines from the American College of Obstetricians and Gynecologists [18] and the American Diabetes Association [19]. We tailored the pregnancy phase of the intervention to the needs of women immediately following a diagnosis of GDM and centered on increasing women’s understanding of the development of GDM, how maternal glucose levels can affect fetal growth and the role of diet and exercise in weight and glucose control during pregnancy. Participants were asked to login weekly to record their weight and fasting glucose levels, and to review web lesson, videos and health tips. Participants were also encouraged to post questions, concerns and successful weight or glucose control strategies on the program’s message board. Participants were sent a weekly text message or email (based on their preference) that included a health tip or motivational phrase to help them achieve weight and glucose control. Participants were sent a text message congratulating them after delivery of the infant was confirmed through hospital records. During the 4^th^ week after delivery, participants were sent a text message that included a health tip and a reminder that the study coordinator would be meeting them at their 6-week postpartum visit to begin the postpartum phase of the intervention. Each participant was contacted by the study coordinator via text, email or phone to confirm the day and time of their postpartum visit.

#### Postpartum phase

The goal of the postpartum phase of the intervention was to help women return to their pre-pregnancy weight or achieve a 5 % weight loss. The postpartum phase began with the 6-week postpartum visit and continued for 24 weeks. The program consisted of 24 web lessons (Appendix) adapted from the DPP [8] and previously delivered using web-based technology [20–23]. Many of the behavioral strategies used in DPP have parallels with the postpartum period, such as skills training in modeling healthy eating behaviors, and balancing time for infant care and family commitments with consistent physical activity. Web lessons were adapted to address some of the unique challenges faced by mothers in the early postpartum period, such as the challenging effects of newborn care on maternal sleep, how to exercise with an infant, and the importance of continuing healthy lifestyle behaviors after delivery to reduce the chances of developing postpartum insulin resistance and hyperglycemia. Participants’ weights were displayed in graphic form by the program so that participants could view their progress over time. As with the pregnancy phase, participants received automated tailored feedback about their weight, physical activity and diet behaviors reported in the weekly website check-in questions. Participants also received weekly text messages and emails with health-related tips and strategies to further assist with reaching their goals. Physical activity goals were the same as those outlined for the pregnancy phase (30 min per week at least 5 times per week).

#### Webinars

Recognizing the need to balance the opportunity for peer support with the logistics of childcare, work and family commitments, we developed and conducted 4 live webinars (2 in the pregnancy phase and 2 in the postpartum) during the intervention. Webinars topics focused on eating habits and strategies to maintain healthy eating during social and work events. Additional topics included strategies for maintaining a regular exercise routine and how to cook healthy meals for their families and themselves. Webinars were led by trained interventionists and were semi-structured, allowing time for presentation of the topic, group interaction and problem solving barriers.

### Measures

The primary outcome measure was feasibility, as determined by rates of recruitment and participant completion of the intervention. Socio-demographics and medical history were collected through self-report at baseline. Information on the use of oral diabetes medications or insulin was based on maternal self-report at the 6-week postpartum study visit. Women were asked to attend an enrollment visit and three follow-up study visits: the enrollment (first) visit occurred within 2 weeks of the diagnosis of GDM and baseline data was collected. The three follow-up study visits occurred at 36 weeks of pregnancy and 6 weeks and 30 weeks postpartum. Two trained research assistants collected baseline and follow-up data.

### Clinical measures

Weight was measured at each study visit with a digital electronic scale. Gestational weight gain (GWG) was calculated as the difference between the weight at 36 weeks of pregnancy and the earliest first trimester weight. We used weight at 36 weeks as the final data collection time point rather than the weight at the time of maternity ward admission in order to minimize the variability in gestational age at the time of the final collection of weight. Earlier studies have used 36 or 37 weeks’ gestation as the time for final weight measures. By using a similar endpoint, we are able to compare our outcome measures to other published data. To avoid the risk of bias in determining pre-pregnancy body mass index (BMI) category, we chose to use measured data from the earliest first trimester visit rather than a participant’s self-reported pre-pregnancy weight. [24] The average weekly rate of weight gain during enrollment was calculated as the difference in weight between study enrollment and 36 weeks of pregnancy divided by the total number of weeks during this same time period. Postpartum weight retention was calculated as the difference in weight at 30 weeks postpartum and the earliest first trimester weight.

### Other measures

#### Laboratory

Hemoglobin A1c (HbA1c) is emerging as a potential tool to predict development of GDM and a useful monitoring tool in pregnancy [25]. Maternal blood was collected in EDTA tubes, immediately inverted several times to prevent coagulation and transported to the institution’s core lab. HbA1c was measured at baseline and at 36 weeks of pregnancy.

In the postpartum phase, fasting maternal blood was collected in serum separator tubes and transported on dry ice to the laboratory for analysis. Fasting glucose was measured with the UV-hexokinanse method on a glucose analyzer (Beckman Diagnostic) at 6 weeks and 30 weeks postpartum. Fasting insulin was measured via radioimmunoassay (Linco Research, Inc). Insulin resistance [26] was assessed, as measured by the homeostasis model of assessment-insulin resistance (HOMA-IR = insulin*glucose/405) [12].

#### Self-reported behaviors

We assessed the feasibility of administering questionnaires at baseline and at each of the 3 follow-up study visits. Self-efficacy in physical activity was assessed using a validated 5-item instrument [27]. min per day of walking in bouts of ≥ 10 min were determined using the Paffenberger questionnaire. Dietary intake was assessed with the Block Food Frequency Assessment [28]. Breastfeeding status was assessed using the infant feeding practices survey (http://www.cdc.gov/ifps/)

#### Infant measures

Because we conducted a multi-site study with different institutions for labor management and delivery, infant birth weight was based on maternal self-report. Infant weight at 6 weeks and 30 weeks after delivery was measured using a calibrated scale (Scale-Tronix, Wheaton, Ill) by study staff. Infant length obtained using an infant measuring board based on standard protocols. Harpenden calipers (British Indicators, Sussex, England) were used for triceps skinfold measurements [29]. Circumference of the head was measured using a measuring tape. Head circumference and skinfold thickness were measured twice and an average measurement was used in the analysis.

##### Acceptability

A post-intervention focus group was used to explore the acceptability of the length and format of the intervention, timing of intervention delivery in relation to the diagnosis of GDM and initiation of the postpartum phase of the intervention after delivery.

##### Statistical analysis

We conducted descriptive analyses (calculated means and frequencies) for socio-demographics, health variables, dietary intake and physical activity. The proportion of women whose gestational weight gain was within the IOM guidelines was assessed across BMI categories using the chi-square statistic. We calculated the change in postpartum weight based on all participants who completed both the 6-week and 30-week postpartum visits. We performed a sensitivity analysis, including all participants who had a 6 weeks postpartum weight, assuming that participant who did not have a 30-week follow-up weight remained at their 6 week postpartum weight. The proportion of participants who met or exceeded their postpartum weight loss goal at 6 weeks and 30 weeks postpartum was compared across BMI categories. Analysis of pre/post changes in minutes/week of moderate walking in bouts ≥ 10 min, A1c levels and HOMA-IR, was performed using paired t-tests. We also analyzed self-reported change in percent fat and fruit and vegetable score and self-efficacy in physical activity. Because depressive symptoms have been shown to affect eating behaviors, we also assessed depressive symptoms using the Edinburgh depression scale. All analyses were conducted using STATA statistical software, version 10.0 (Stata Corporation, College Station, TX).

## Results

### Feasibility

**Fig. 1:**
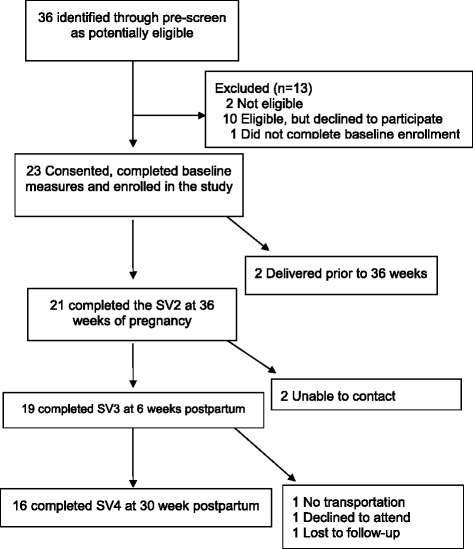
Participant flow in GooDMoMs feasibility study

Of the 36 women who were screened for eligibility, 23 (64 %) were eligible, agreed to participate and completed baseline assessments (Fig. 1). Thirteen women were excluded because they were ineligible (*n* = 2), eligible, but declined to participate (*n* = 10) or did not complete the enrollment process (n-1). Retention was 91 % at the 36-week follow-up visit and 83 % and 70 % at 6 weeks and 30 weeks postpartum, respectively.

### Website use

Website logins were recorded by the GooDMomS system. Sixty-five percent (15/23) of the participants logged into the website at least 3 times during the pregnancy phase of the intervention. Ten of the 16 participants (62 %) who completed the postpartum phase of the intervention logged into the system at least once during the postpartum period; six participants had 10 logins during the phase.

### Demographics

Average maternal age at enrollment was 31.7 years (SD = 4.7). Of the 23 participants, 16 were Caucasian, 3 were African American, 3 Asian and 1 reported their race/ethnicity as “other” (Table 1). The racial distribution of the participants reflected the demographics of the two clinics included in the study. Most participants were married, had a college degree or attended some college, were employed at the time of enrollment, and had commercial insurance. Almost half of the participants were nulliparous. All participants reported internet access for at least 4 h per week. The median gestational age at enrollment was 28.9 weeks. Average first trimester BMI was 29.4 kg/m^2^. Nine participants had a normal BMI, 4 were overweight and 10 were obese. Average weight at enrollment was 174.4 lb (SD = 41.4). There were no significant differences between women included in the study and women who were ineligible or declined to participate with regard to age, race/ethnicity, gestational age or parity. There was no difference in age, race or parity among women who completed study visits compared to those who did not complete the study.

**Table 1:** Demographic and clinical characteristics of participants at baseline, *N* = 23

Socio-demographics	
Age, (yrs.), mean (SD)	31.5 (4.7)
Race	
White	16 (70)
African American	3 (13)
Asian	3 (13)
Other	1(4)
Hispanic	4 (17)
Education	
High school or less	5 (22)
Some college/complete college degree	12 (52)
Graduate/post graduate	6 (2.6)
Marital status	
Married	19 (83)
Single	4 (17)
Employed	14 (61)
Source of medical payment	
Commercial	18 (78)
Medicaid	2 (9)
Self-pay	2 (9)
Medicare	1 (4)
Parity, n, no prior delivery	11 (48)
1	5 (22)
2 or more	7 (30)
First-degree family history of diabetes	5 (22)
Smoking before pregnancy	23 (100)
Gestational age at baseline, (wks), mean (SD)	28.9 (3.4)
Pre-pregnancy BMI^a^ (kg/m^2^)	29.4 (11.2)
Normal (18.5–24.9)	9 (39)
Overweight (25.0–29.9)	4 (17)
Obese (30–34)	10 (43)
Earliest first trimester weight (kg), mean (SD)	71.7 (18.2)
Weight at enrollment, (kgs), mean (SD)	79 (18.8)
Gestational weight gain at enrollment (kgs), mean (SD)	7 (5)
Therapy during pregnancy after GDM diagnosis	
Diet	16 (70)
Glyburide or metformin	6 (26)
Insulin	1 (4)
Internet access at least 4 h per week	23 (100)
1-h 50-g glucose challenge test (mg/dl), mean (SD)	155 (10)
3-h, 100-g OGTT (mg/dl), mean (SD)	
Fasting	100 (5)
1-h	183 (30)
2-h	165 (25)
3-h	145 (15)
% dietary intake at baseline	
Percent fat (%), mean (SD)	36 (5)
Fruit and vegetable (servings/day), mean (SD)	3.2(1.6)
Moderate walking in bouts ≥ 10 min (minutes/week) mean (SD)	159 (185.7)

*Kg* kilograms; Data are reported as n (%) unless otherwise indicated

^a^Pre-pregnancy BMI is based on weight and height measured at earliest first trimester visit

### Clinical outcomes

#### Gestational weight gain

Average gestational weight gain for all participants was 19.9 lbs ± 13.2. About three-quarters of participants were within the IOM guidelines for gestational weight gain at 36 weeks of pregnancy (Table 2). Fifty percent of women in the overweight or obese categories were within the IOM guidelines for weight gain. All of the participants with a normal BMI were within the IOM guidelines for weight. The average weekly rate of weight gain during the intervention period is summarized in Table 2. Half of the obese participants had an average weekly weight gain rate within the IOM guidelines; 78 % and 67 % of normal and overweight women, respectively, had a weekly rate of weight gain with the range recommended by the IOM.

**Table 2:** Gestational weight gain of women with GDM and proportion meeting the IOM weight gain goals at 36 weeks of gestation

	All participants	Pre-pregnancy BMI^1^		
Weight gain variables	*N* = 21	Normal *n* = 9	Overweight *n* = 4	Obese *n* = 8
Total gestational weight gain, kgs, mean (SD)	9 (5.9)	10.3 (2.8)	7.7 (12)	6.9 (6.6)
Proportion with gestational weight gain within IOM^2^ guidelines, n (%)	15 (71)	9 (100)	2 (50)	4 (50)
Gestational weight gain during GooDMomS intervention, lbs, mean (SD)	1.8 (2.1)	1.3 (1.9)	0.7(1.0)	2.4 (2.5)
Average number of weeks in intervention, mean (SD); range	6.6 (3) (2–12)	6.4(2.9) (2–11)	6 (2.0) (4–11)	7.1 (3.6) (2–12)
Average weekly rate of weight gain^3^ during GooDMomS intervention	0.4(0.7) (−0.7, 2.7)	0.2 (0.4) (−0.7, 0.6)	0.2 (0.3) (−0.05,0.5)	0.6 (1.0) (−0.01, 3.2)
Proportion within 3^rd^ trimester recommended weekly rate of weight gain during intervention, n(%)	13 (62)	7 (78)	2 (67)	4 (50)

*GDM* gestational diabetes mellitus, *IOM* Institute of Medicine. ^1 ﻿^Pre-pregnancy BMI categories (kg/m2) are based on weight measured at earliest first trimester prenatal visit: normal (18.5–24.9); overweight (25.0–29.9); obese (30 or greater)

^2^Gestational weight gain by IOM guidelines: normal BMI (11.3–15.9 kgs); overweight (6.8–11.3 kgs); obese (5–7.3 kgs)

^3^Weekly rate of weight gain (kg/week) by BMI category: normal [0.5 (0.4–0.5)]; overweight [0.3 (0.2–0.3)]; obese: [0.2 (0.2–0.3)]

### Postpartum weight and weight retention

Participants achieved weight loss during the postpartum phase of the intervention (Table 3). Average weight loss between 6 and 30 weeks postpartum was 6.4 ± 52 lbs. Body mass index decreased by 2.6 kg/m^2^ during the same time period. Average weight retention was 2.14 ± 15.2 and 0.9 ± 12 at 6 weeks and 30 weeks postpartum, respectively. Among participants with a normal pre-pregnancy BMI, 7/16 had met or exceeded their weight loss goal at 6 weeks postpartum; 9/16 had met or exceeded their weight loss goal by 30 weeks postpartum. Among overweight/obese participants, 6/8 (75 %) had returned to their pre-pregnancy weight at 6 weeks. At 30 weeks postpartum, 4/8 (50 %) overweight/obese participants met or exceeded their weight loss goal (percent change −25 %; *p* = 0.3). Two of the eight participants gained weight between the 6 weeks and 30 weeks postpartum visit.

**Table 3:** Postpartum weight retention and proportion of women with GDM meeting or exceeding weight goals^1^

	6 weeks PP *n* = 16	30 weeks PP *n* = 16	Mean change,^2^ *p*-value
Postpartum weight (kgs), mean ± sd	72.6 ± 18	69 ± 18.1	−2.9 ± 23.6 *p* = 0.6
Postpartum weight retention (kgs), mean ± sd	1.0 ± 6.9	0.4 ± 5.9	−0.5 ± 9.1; *p* = 0.8
Proportion meeting/exceeding weight goal^3^, n (%)			Percent change,^3^ *p*-value
All participants	7/16 (43 %)	9/16 (56 %)	13 %’; *p* = 0.4
Normal BMI (18.5–24.9 kg/m^2^)	1/8 (12.5 %)	5/8 (62.5 %)	49 %; *p* = 0.03
Overweight/obese BMI (25–34 kg/m^2^)	6/8 (75 %)	4/8 (50 %)	−25 %; *p* = 0.3

*GDM* gestational diabetes; kg = kilograms; sd = standard deviation; PP = postpartum

^1^Values are based on the 16 participants with complete weight data at 6 weeks and 30 weeks postpartum

^2^mean change represents the mean difference in weight retention at 6 weeks and 30 weeks postpartum

^3^postpartum weight goal was a return to the weight at the earliest first trimester visit or a 5 % weight loss, whichever was greater

represents percentage change in the proportion of participants meeting or exceeding their weight loss goal

### Other outcomes

The majority of participants reported walking as their primary source of moderate physical activity. Participants reported an average of 159 ± 185.7 min of walking/week at baseline, 94 ± 83.4 min/week at 36 weeks, and 88.5 ± 153.3 min/week and 77.4 ± 123 min/week at 6 weeks and 30 weeks postpartum, respectively. There was no difference in self-efficacy scores for physical activity during pregnancy. However, scores were lower at 30 weeks postpartum compared to enrollment (mean change: −2.3; [−4.3, −0.2]; *p* = 0.3)

Daily fat intake decreased from baseline; fruit and vegetable consumption increased during the intervention period. Of the 16 participants that presented to the 6-week postpartum study visit, 12 participants were exclusively breastfeeding, one was partially breastfeeding and 3 were using formula. At 30 weeks postpartum, 10 participants were breastfeeding exclusively and 3 were partially breastfeeding. Two participants had depressive symptom scores above 12 (scores were 13 and 14) at baseline and were referred for counseling. Average depressive symptoms scores ranged from 5.9 ± 3.8 to 3.1 ± 3.7 at 30 weeks postpartum.

There was a small increase in mean A1C levels from baseline (5.08 ± 0.22) to 36 weeks’ gestation (5.13 ± 0.16), but the change was not statistically significant (mean difference: 0.13; *p* = 0.5). Insulin resistance, as measured by HOMA-IR increased between 6 weeks (2.3 ± 2.5) and 30 weeks (14.1 ± 21) postpartum period. Only 8 (50 %) of the 16 women participants who attended the 30 weeks postpartum visit were in a fasting state.

#### Infant measures

Of the 19 mothers presenting for the 6 week postpartum visit, 19 (100 %) infants were available for assessment at 6 weeks postpartum. Average weight at 6 weeks 4.0 ± 0.5 and triceps skinfold thickness was 8 ± 2.7 mm. Of the 16 participants presenting for the 30 week visit, nine (56 %) women also brought their infants for the newborn assessment. Average weight was 7.4 ± kg 1.2 and triceps skinfold thickness was 10.2 ± 3.7 mm.

### Post-intervention focus group

We conducted a post-intervention focus group with 10 women who completed the intervention to gain feedback on 1) the usefulness of the intervention materials on the website, text messages and emails, 2) barriers to logging into the GooDMomS website and 3) perceived challenges to achieving their weight goals and glucose control. Participants thought that the intervention materials during pregnancy were useful and provided a better understanding of how gestational weight gain during pregnancy can affect the growth of the infant. The weekly emails and text messages during pregnancy and postpartum were well received by participants. In terms of intervention delivery, three themes emerged: 1) the need for a formal transition to the postpartum phase of the intervention with an emphasis on postpartum goals and objectives, 2) modifications to the website to notify participants when someone has posted a question or message on the message board to promote better interaction among participants, and 3) transition to a mobile application. Women expressed that logging into the system using a desktop or laptop computer during the postpartum phase was challenging due to the demands of childcare and that a mobile phone accessible intervention would enhance their ability to login consistently.

## Discussion with lessons learned

In Phase 1, we were able to engage women with current or recent GDM to inform refinements to the GooDMomS intervention for the feasibility study in Phase 2. Our results show that a web-based, pregnancy and postpartum behavioral intervention for women with GDM is feasible. We were able to adapt ACOG [18] and ADA [19] guidelines for glucose and weight management during pregnancy and the DPP lifestyle intervention [8] to develop a pregnancy and postpartum intervention for women with GDM and their offspring. Our overall retention rate of 70 % at 6 months postpartum is acceptable. Collection of self-reported lifestyle behaviors using standardized questionnaires was also feasible. Of the participants who completed the study visits, we were able to collect questionnaires on 100 % (enrollment, visit 1), 80 % at visit 2, and 87 % and 81 %, respectively, at study visits 3 and 4. The Block Food Frequency questionnaire [28] required additional time during study visits and was burdensome to some participants.

During the pregnancy phase, the majority of participants were able to stay within the IOM guidelines for gestational weight gain. The intervention helped women to meet their postpartum weight loss goals by reducing postpartum weight retention in normal weight women and helping overweight and obese women lose weight. It is possible that postpartum weight loss was greater than 6.4lbs, as we used a fairly conservative sensitivity analysis to assess postpartum weight that included only those participants with weight data at both 6 weeks and 30 weeks after delivery.

It was challenging for participants to reach the physical activity goal outlined in the intervention. Despite web lessons that focused on the benefits of moderate physical activity, there was a decline in moderate walking during pregnancy and postpartum. It may be that some participants had lingering concerns about the risks of exercise during pregnancy, although none of the participants had contraindications to physical activity. Further refinements to the web lessons to emphasize the safety of exercise in pregnancy and postpartum may improve participants’ ability to reach their physical activity goals.

This study contributes to the care of women with GDM in several important ways. First, we refined and introduced an intensive web-based lifestyle intervention integrated seamlessly across two critical periods in the lifespan of both mother and child – the prenatal period following the diagnosis of GDM and the metabolic alterations that occur in women and their offspring in the postpartum period. Prior studies of behavioral interventions in women with GDM have largely been confined to the pregnancy/delivery period [4], the postpartum timeframe alone [6,30], or on early childhood growth following delivery [31–33]. Ferrara and colleagues [5] assessed pregnancy and postpartum intervention, that was conducted using in-person sessions and telephone contacts. To our knowledge, GooDMomS is one of the first pregnancy and postpartum interventions delivered via web-based technologies. Second, this study adds to the literature on behavioral interventions that can be successfully delivered to pregnant and postpartum women, as the feedback from the post study focus group indicated that the intervention interface and content was both accessible and useful [34]. National studies and investigations within our institution [35] confirm access to and use of web-based technologies among pregnant women. Third, this study represents one of the first efforts to measure the preliminary effects of a web-supported behavioral intervention that begins with the diagnosis of GDM, continues through pregnancy and postpartum and include weight and body composition measures of the offspring.

Strengths of this study include our mix-methods approach. We used qualitative methods to refine the intervention in phase 1 and quantitative methods to test feasibility in phase 2. We also engaged women who had participated in the feasibility study in meaningful conversations to gain feedback and insights on the GooDMomS intervention. Participants reported that the intervention was acceptable and useful during the pregnancy and postpartum phases of the program. With the functionality of the system, we were able to directly assess logins rates rather than relying on participant self-report as a measure of utilization. Further refinements to GooDMomS that facilitate its use on smartphones or as a mobile app may improve utilization.

There are limitations to our findings. The small sample size and pre/post design in phase 2 was appropriate to assess feasibility, but a larger scale controlled study is needed to determine effect sizes and confirm the results. This feasibility study helped to inform specific refinements to the intervention that can help to improve acceptability by future participants. Also we were able to confirm the viability of our recruitment strategies as well as the identification and enrollment of participants. Participants represented a convenience sample of women attending a tertiary care prenatal care clinic, which limits generalizability of the findings to women obtaining care in other types of health care settings.

Several lessons were learned from this feasibility study. The follow-up rate at the 30-week postpartum study visit (70 %) was lower than expected. Limited transportation and work commitments were barriers to follow-up at the 30-week visit. Additionally, participants faced some difficulties in transporting their newborns to the 30-week visit and then back to their place of childcare. In future studies, it may be more effective to schedule postpartum follow-up visits to correlate with well child visits and arrange for data collection to take place within the pediatrician’s office. As such, we may be able to improve long-term retention and enhance data collection. It was also challenging for women to present in a fasting state at the postpartum visits. Our study experience was similar to reports that have documented the low rates of compliance with postpartum glucose testing in women with recent GDM. Providing an opportunity for participants to complete the postpartum testing on a weekend may improve compliance. Regarding the intervention, postpartum login rates were lower than in the pregnancy phase of the study. Additional text messages to remind participants to log into GooDMomS as well as a re-introduction to the goals and objectives of the postpartum phase of the program at the 6 weeks postpartum study visit could enhance postpartum login rates. Family commitments and the demands of infant care were verbalized by participants in the post-intervention focus groups as contributing to the lower login rates and less adherence to the study protocol. Further work is needed to determine the best methods to promote consistency in healthy behaviors and website logins after delivery. Behavioral interventions that promote lifestyle change are critical to reducing perinatal obesity, hyperglycemia and the risk of type 2 diabetes.

## Conclusions

Our study suggests that a web-based behavioral intervention combined with text messages and emails and tailored to the needs of women with GDM is feasible and well received by participants. Study results show that GooDMomS can help women stay within the IOM guidelines for gestational weight gain and achieve their postpartum weight loss goals. GooDMomS can potentially play an important role in changing the current paradigm of pregnancy care for women with GDM. More importantly, the program may serve to fill an important gap in care after delivery, particularly with regard to physical activity and postpartum weight loss. Further study is needed in a larger sample of participants with short and long-term clinical outcome measures in mothers and infants.

## Appendix

**Table 4:** Gestational Diabetes Management Systems Web Lessons

Pregnancy Web Lessons and videos	Postpartum Web Lessons
1. Gestational Diabetes – The Basics	1. Getting Started
2. Video Lesson #1: How did I get Gestational Diabetes	
3. Treating Your Gestational Diabetes (Part A)	2. Energy Balance
4. The Benefits of Exercise for Women with Gestational Diabetes	3. Daily Weighing
5. Video Lesson #2: Benefits of Exercise for you and your baby	4. Becoming More Active
6. Treating Your Gestational Diabetes (Part B)	5. Incorporating Physical Activity into Your Everyday Life
7. Benefits of Breastfeeding for You and Your Baby	6. Be a Fat Detective
8. Preparing for postpartum Glucose Testing	7. Eating Out on a Calorie Budget
9. Video Lesson #3: Getting ready for your postpartum visit and glucose testing	8. Feeding Your Family
	9. Social Support
	10. Take Charge of What’s Around You
	11. Cues for Activity
	12. Simple Ways to Control Calories
	13. Recipe Modification
	14. Emotional Overeating
	15. Barriers to Diet and Exercise – Fatigue
	16. Time Management
	17. Problem Solving
	18. Meal Planning
	19. Stress Management
	20. Eating Patterns
	21. Thoughts and Weight Control
	22. Maintaining Motivation
	23. Staying in Control of Your Weight
	24. Becoming a Weight Control Expert
